# The Parental and Children Report of the Prevalence of Depressive Symptoms in Children and Adolescents Amid the COVID-19 Pandemic: A Cross-Sectional Study From Oman

**DOI:** 10.3389/ijph.2022.1604474

**Published:** 2022-08-25

**Authors:** Fahad Zadjali, Amna Al-Futaisi, Amira Al-Hosni, Salim Al-Huseini, Maarten Crommelin, Hassan Mirza

**Affiliations:** ^1^ Department of Biochemistry, College of Medicine and Health Sciences, Sultan Qaboos University, Muscat, Oman; ^2^ Department of Child Health, College of Medicine and Health Sciences, Sultan Qaboos University, Muscat, Oman; ^3^ Department of Behavioural Medicine, Sultan Qaboos University Hospital, Muscat, Oman; ^4^ Psychiatry Residency Program, Oman Medical Specialty Board, Muscat, Oman; ^5^ South London and Maudsley NHS Foundation Trust, London, United Kingdom

**Keywords:** COVID-19, pandemic, psychological impact, adolescent psychiatry, children, depression, symptoms, Oman

## Abstract

**Objective:** Studies from the past decades have shown that mood disorders are common during childhood and adolescence. This study aimed to estimate the point prevalence of depression in Omani children and adolescents during social distancing and lockdown and identify the risk factors for developing depressive symptoms during the COVID-19 pandemic.

**Methods:** This is an analytical cross-sectional study conducted in May 2020, in which all young Omani people attending a mainstream school aged 8–18 years old were eligible to participate. Parents were asked to complete the online survey, which consisted of the parent version of the Mood and Feelings Questionnaire (MFQ-Parent). In addition, the option of a self-reported version (MFQ-Self) was provided in cases where children preferred to fill out the survey themselves. Logistic regression was used to identify the contributing socio-demographic variables associated with depressive symptoms.

**Results:** A total of 445 participants completed the MFQ, out of which 72.1% were parents, and 27.9% were children, adolescents and young people. 13.9% of children and adolescents exhibited depressive symptoms during the COVID-19 pandemic in Oman. The presence of depressive symptoms was associated with increased food intake (OR 1.81, 95% CI 1.00–3.29, *p*-value <0.05), longer use of smartphones (OR 2.72, 95% CI 1.56–4.73, *p*-value <0.001), whereas additional entertainment activities during lockdown were protective against depression (OR 0.35 95% CI 0.19–0.64, *p*-value <0.001).

**Conclusion:** This study from Oman concurs with recent reports of depression being common during the COVID-19 pandemic. Concerted efforts are needed to mitigate this trend and identify high-risk groups during the lockdown period.

## Introduction

Mood disorders are disabling mental disorders that negatively impact all function domains, ranging from impairment in work adjustment, family and personal relationships to more severe deterioration resulting in severe morbidity and disability [1]. The diagnostic criteria for depression in children are the same as in adults; this includes persistent low mood, loss of interest, poor concentration, fatigue, changes in sleep and appetite, associated with a feeling of worthlessness, hopelessness, and potential thoughts of suicide [2].

Not too long ago, mood disorders such as depression were considered adult disorders. In contrast, children were considered too developmentally immature to experience the core symptoms of clinical depression [3]. However, research development over the past decades has shown that mood disorders are common during adolescence, resulting in a higher risk of self-harm, suicide, poor school performance, and substance abuse [4]. Nevertheless, diagnosing mood disorders in young people may have its challenges. For example, there can be a delay in diagnosis due to atypical presentation, resulting in a poorer prognosis and lower likelihood of recovery [5]. Suicide is the second leading cause of death in adolescents, with more than 50% of adolescent suicide victims having reported clinical depression at the time of death [6], making depression a significant and major risk factor and cause of suicide in young people [7, 8]. Although the exact cause of mood disorders is not very well understood and difficult to pinpoint, researchers believe that genetic, environmental, and psychological factors play a major role in their development [9]. Similarly, theories involving an imbalance of neurotransmitters, such as serotonin and norepinephrine, have been hypothesized to be factors in the development of mood disorders [10]. A study from Oman looked into the prevalence of mental disorders among school going Omani adolescents found that the estimated lifetime prevalence of major depressive disorder in young people was 3% [11]. In contrast, a systematic review and meta-analysis examined the global prevalence of depression in adolescents from 2001 to 2020 concluded that the point prevalence rate of depression was 8%, with adolescents from Asia, Africa, and the Middle East having the highest risk of developing depression [12]. Similarly, a meta-analysis on the prevalence of depressive symptoms during the COVID-19 pandemic among adolescents showed that the pooled prevalence estimates of clinically elevated depressive symptoms in young people were approximately 25% [13]. Furthermore, a large study looked into self-harm and inpatient admissions among children and adolescents from 10 different countries, including Oman [14], the findings had significant implications for service planning during a possible future wave of COVID-19 or a lockdown for any other public health emergency. The emergence of the COVID-19 pandemic has led to many changes globally. The cancellation of all academic and social events, the closure of schools, shops, and the implementation of social distancing has resulted in immense stress for the general population. Oman implemented social distancing early on in March 2020, which resulted in the suspension of schools, with some institutes introducing online classes for their students (“COVID-19 Supreme Committee Holds First Meeting,” 2020) but not all. Outbreaks in the past, including SARS, Ebola and the H1N1 swine flu, had a mental health impact on the general population, causing anxiety, depression, and post-traumatic stress [15]. A large-scale nationwide survey of psychological distress in the general population of China during the COVID-19 pandemic showed that a substantial proportion experienced psychological distress [16]. Similarly, a study looking into the psychological effects of lockdown on students in a Spanish university reported high scores related to depression, anxiety and emotional difficulties [17]. Moreover, a systemic review of the impact of quarantine and its psychological ramifications reported a wide range of mental health-related difficulties, which may have long-lasting effects [18]. Furthermore, numerous studies have looked into the association of the frequency of physical activity, sedentary lifestyle, and the development of depression in the young [19, 20]. Therefore, exploring these factors in the emergence of depressive symptoms in the young population during the lockdown is crucial.

Screening for depressive symptoms during this period will help us understand the psychological impact of lockdown, homestay, and social distancing whilst identifying any potential service recommendations for future lockdowns and emergencies. We should use this time to gauge the emerging mental health burden on the young and set up a concrete foundation for tailoring relevant mental health services to mitigate the psychological impact of future pandemics efficiently and effectively. Identifying the factors contributing to decreased depressive symptoms in children and young adolescents provides insights into the early management of any psychological impact experienced from being in lockdown or homestay. In this study, we aimed to assess the prevalence of depressive symptoms in children and adolescents during social distancing and to identify risk factors associated with these symptoms.

## Methods

This is a web-based cross-sectional analytic study carried out during May 2020. All young Omani people attending a mainstream school aged 8 to 18 were eligible to participate. Google forms were sent through email, WhatsApp, and Twitter to the general public, and the survey remained open for 1 week. The Google form links were sent to the parents’ email and WhatsApp, and it contained the option of the parent version and the self-reported version. Young people with access to social media platforms that filled the self-reported version had to get parental consent to participate. However, parental approval verification was difficult, considering the survey was not face-to-face. All the answers were combined for analysis, and parents with more than one child aged 8–18 had the option to fill out a new form.

We based our sample size on a previous study about the prevalence of depression in attendees of urban primary healthcare centres in Oman, using the patient health questionnaire (PHQ). Where he reported prevalence of depressive symptoms was 8.1% [21]. With a 95% confidence interval, a 2.5% margin of error, and a population size of 3 million, the minimum sample size for the survey was calculated as a total of 453. The research tool consisted of an online questionnaire which comprised two parts. The first section included the following sociodemographic and risk factor variables: age, gender, and place of residence, number of siblings and rooms in the house, attendance of online schooling, change in food intake and physical activity, introduction and practice of new entertainment activities, frequency of use of electronic games and smartphones and whether any family members tested positive for COVID-19. The second part consisted of an adapted version of the short child and parent Mood and Feelings Questionnaire (CMFQ and PMFQ, respectively), which was developed to measure depression probability in child and adolescent populations. It captures cognitive, affective, vegetative and suicidal aspects of depression. It has been validated in both English and Arabic [22, 23]. The questionnaire provides a rapid assessment for depressive symptoms in epidemiological and clinical settings. We have obtained permission to use MFQ from the primary authors and used a cut-off value of 12 and above as a definition of depressive disorder [24, 25].

We used google forms to generate the questionnaires and distributed links to parents using email and social media platforms. Instructions provided for the completion of the questionnaire include the requirement that children must obtain the permission and consent of parents before completing the questionnaire. Microsoft Excel was used to perform basic statistics, and an R-package forest plot was used to generate an unadjusted odds ratio for multiple risk factors. A *p*-value of <0.05 was adopted for statistical significance.

### Internal Reliability and Validity of the Mood and Feelings Questionnaire

Internal reliability was assessed separately by examining the Cronbach’s alpha scores of the short MFQ for parents (PMFQ) and children or young adolescents (CMFQ). According to Ponterotto and Ruckdeschel’s [26] classifications, Cronbach’s alpha values for both MFQs were excellent (α = 0.91). Each item had strong reliability and no drops in Cronbach’s alpha values with item deletion. The corrected item whole correlation score examined content validity. Both PMFQ and CMFQ items had good correlations with average values of 0.67 and 0.68, respectively (Table 1). Item Q13 (“the child did everything wrong”) had the lowest correlation of 0.54 for PMFQ and 0.58 for CMFQ. The correlation and Cronbach’s alpha scores were similar between the parent and child MFQs, suggesting excellent convergent validity in the questionnaire.

**TABLE 1:** Mood and Feelings Questionnaire reliability and content validity (Muscat, Oman. 2020).

	MFQ-parent		MFQ-child	
	Correlation	Alpha-drop	Correlation	Alpha-drop
1. I felt miserable or unhappy	0.62	0.91	0.72	0.91
2. I didn’t enjoy anything	0.72	0.9	0.59	0.91
3. I felt so tired that I just used to sit down without doing anything	0.69	0.9	0.63	0.91
4. I felt very agitated	0.72	0.9	0.77	0.91
5. I felt worthless	0.71	0.9	0.73	0.91
6. I cried a lot	0.61	0.91	0.71	0.91
7. It was hard to think or to concentrate	0.73	0.9	0.7	0.91
8. I hated myself	0.73	0.9	0.77	0.91
9. I was a bad person	0.6	0.91	0.63	0.91
10. I felt lonely	0.69	0.9	0.73	0.91
11. I thought that nobody loved me	0.64	0.91	0.64	0.91
12. I thought that I would never be as good as other children or adolescents	0.73	0.9	0.64	0.91
13. I did everything wrong	0.54	0.91	0.58	0.91
**Average**	**0.67**	**0.90**	**0.68**	**0.91**

Item whole correlation corrected for item overlap and scale reliability.

### Ethical Approval

Ethical approval was granted by the College of Medicine and Health Sciences at Sultan Qaboos University, Muscat, Oman (MREC 2105/20). The study was conducted per the Declaration of Helsinki and the American Psychological Association regarding human ethical research, including confidentiality, privacy and data management.

## Results

### Study Subject Characteristics

A total of 445 respondents completed the MFQ, of which 72.1% were parents, and 27.9% were children, adolescents or young people (Table 2). All surveys received were complete with no missing items. COVID-19 was more widespread in the capital city of Muscat, where an early regional lockdown was implemented. Most of the study participants were inhabitants of the capital city of Muscat (57.6%). Nuclear families in Oman are relatively large, and 56.1% of subjects had between 2–5 children or siblings, 41.0% had 7–12 rooms in the house. Strict social distancing was practised by 65% of study subjects during the homestay imposed by the health committee.

**TABLE 2:** Characteristics of study subjects (Muscat, Oman. 2020).

	PMFQ	CMFQ	MFQ-total
N (%)	321 (72.1)	124 (27.9)	445
Male%	38.6	42.5	39.9
Age (year)	38.0 + 7.8	13.4 + 2.8	
Capital city%	58.3	56.5	57.6
Employed%	70.7	—	—
No. of children/adolescents at home%			
1 child/adolescent	39.1	35.5	38.1
2–5 children/adolescents	55.6	57.3	56.1
6–10 children/adolescents	3.4	4.0	3.6
10 or more children/adolescents	1.6	3.2	2.0
No. of rooms in the house%			
up to 6 rooms	39.8	25.8	35.9
7–12 rooms	37.3	50.8	41.0
>12 rooms	1.9	7.3	3.4
Social distancing strictly applied%	65.1	65.3	65.0
Anyone diagnosed with COVID-19%	0.9	0	0.6
Increased food intake%	71	75.8	72.4
Decreased physical activity%	23.1	18.5	21.8
Online schooling for students provided%	59.2	56.5	58.4
Additional home entertainment activities provided%	72	63.7	70
MFQ score	4.6 + 4.9	7.6 + 6.7	5.5 + 7.1

Data are shown as percentages (%) or mean + SD.

### Prevalence of Depressive Disorder in Children and Adolescence and Its Associated Factors

The MFQ provides a validated assessment of depression probability in child and adolescent populations, capturing cognitive, affective, vegetative and suicidal aspects of depression. We used the combined MFQ of both parent and children MFQ data. We used an MFQ cut-off of 12 and found 62 (13.9%) children or adolescents with a high probability of depressive disorders during the COVID-19 pandemic in the study population.

**FIGURE 1:**
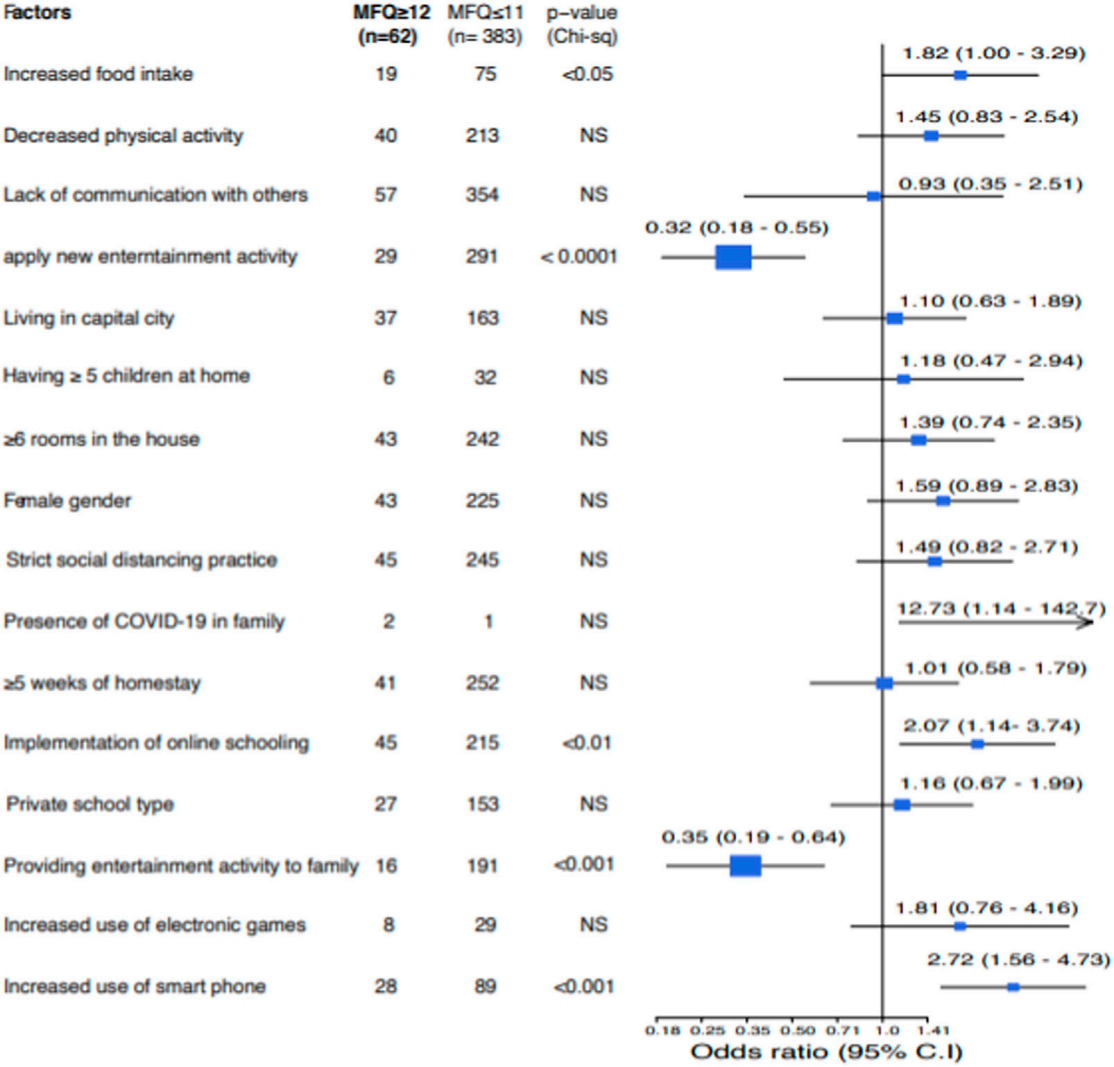
Factors associated with depressive symptoms in children and young adolescents. Subjects with MFQ score of 12 and above are identified with depressive symptoms (Muscat, Oman. 2020). Data are shown as numbers and Chi-square test p-value is shown. Forest plot (right) shows the odds ratio and 95% confidence intervals.

We then assessed the factors contributing to depressive disorder in children and adolescents. There was no association between depressive disorder and gender (Figure 1). Also, the homestay duration (below 5 weeks or more than 5 weeks) was not associated with depressive disorders. We found that depressive disorders during COVID-19 were associated with increased food intake (OR 1.81, 95% CI 1.00–3.29) and increased use of smartphones (OR 2.72, 95% CI 1.56–4.73). These activities could be associated with an increase in depressive symptoms. During the COVID-19 pandemic, restrictions were implemented on meeting people and performing grouped physical activities. We found that lack of communication, reduced physical activities, the number of siblings and room numbers were not associated with depressive disorders in children. Interestingly, we found the odds of having depressive disorders in children and young adolescent is 75% less in those where parents provided additional entertainment activities during the homestay. Here the odds-ratio was 0.35 (95% CI 0.19–0.64). Similarly, we asked if the children or adolescents engaged in these new activities. Positive engagement appeared to be a protective factor with an odds ratio of 0.32 (95% CI 0.18–0.55). Only three reports indicated a family member with COVID-19 infection, and the significance of this could not be accurately calculated due to the small number in the group.

## Discussion

This study emerged that 13.9% of children and adolescents exhibited depressive symptoms during the COVID-19 pandemic. This rate is higher than what is typical in children and adolescents. The prevalence of depression in children and adolescents in the general population is in the range of 2%–8% [27]. In adolescents, the estimated prevalence of depression in the middle to late adolescence is around 4%–5% [28], and studies confirm that it is higher among the female gender, especially in the post-adolescent age group [29].

A study of more than 8,000 Chinese children and adolescents using the Patient Health Questionnaire (PHQ-9) and the Generalized Anxiety Disorder (GAD-7) questionnaire showed that symptoms of depression and anxiety, and a combination of depressive and anxiety symptoms showed a prevalence of 43.7%, 37.4%, and 31.3%, respectively, during the COVID-19 pandemic [30].

There has been a global rise in mental health disorders during the current pandemic. A study from China showed that depressive symptoms during the COVID-19 epidemic are higher among children than adults [31]. Notably, the current study has shown a higher prevalence of depressive symptoms among children and young adolescents during the pandemic. However, this rate seems to be lower than the collective observation among Chinese children. This difference is explainable through the smaller number of participants in the present study than the large-scale studies in China. In this cohort, there is no observable gender difference when assessing factors associated with the presence of depressive symptoms. In general, females are more likely to develop depressive symptoms than males [32]. A previous study on depression among Omani children found that no gender differences exist in the total mean scores of depression [33]. Notably, it appears that several factors may contribute to the lack of gender differences in depressive symptoms. These factors include cultural differences in reporting depressive symptoms, study design, and sample size.

From the study, around 65 per cent of the children and adolescents reported practising strict social distancing measures. This high proportion is commendable and reflects children and families’ high discipline and commitment to the measures implemented to reduce the risk of disease transmission. A study from India looked at children and adolescent compliance with quarantine measures and found low compliance rates (7.43%). They also found better compliance with community protective measures (17.35%) than household protective measures (10.71%) [34]. Besides that, food intake increased by a margin of 72.4% among the participating children and adolescents. There was, however, a decrease in physical activity by 21.8 per cent. A recent study found that during this pandemic, the prevalence of physically inactive students dramatically increased from 21.3% to 65.6%, along with a substantial increase in screen-time to an average of nearly 30 h per week [35].

It became clear that reduced physical activity and prolonged sedentary behaviour are strongly associated with poor mental and physical health outcomes [36]. Physical activity in childhood and adolescence is also linked with improved concomitant symptoms of depression, especially regular and robust physical activity [37]. Although this association is weak, and it has substantial effects on future depressive symptoms. Similarly, studies have shown that young people with overeating and binge eating are more likely to develop depressive symptoms [38].

The use of smartphones was positively associated with depression in our cohort, with an odds ratio of 1.81. This is in line with findings that imply that depression, anxiety, suicide, and inattention among children and adolescents is made grievous by increased screen size time [39]. Another significant finding was that overall screen time is associated with depression and suicidal behaviour among adolescents [40]. A nonlinear dose-response association between depressive symptoms and total screen time is evident among children aged 5 to 18 who were using electronic devices for over 2 hours per day [41].

Other factors such as reduced physical activity, lack of communication with others, the number of siblings, and the number of rooms in the house, confinement, and length of homestay did not have any statistically significant effects on depressive symptoms. Conversely, introducing new entertainment activities in the family positively impacted depressive symptoms and was associated with lower depressive scores. Research confirms that homestay has a chance of improving the prospects of better home interaction between parents and children, improving children’s participation in family activities, and hastening the development of their self-sufficient skills [42]. The protective effect seen with the implementation of new entertainment activities in the family highlights the positive outcomes obtained from close family interactions and the need for families to divert children’s attention towards a more positive and productive direction [43].

Additionally, it emerged that diverse parenting styles and approaches are essential in strengthening family bonds and meeting the psychological needs of children and adolescents [44]. The utilization of new entertainment activities as a tool to lessen the effects of confinement on children and adolescents resulted in fewer depressive symptoms, reflecting children’s appreciation of such gestures from parents, and how these simple strategies immensely helped decrease the psychological impact of this pandemic on children and adolescents. Other measures may include exploring any fears held by children and adolescents, playing collaborative games together, finding creative ways to encourage physical activities, and enjoying music to reduce stress, anxiety, and loneliness associated with confinement and prolonged homestay [42].

Although cross-sectional surveys and self-reported symptoms were imperative and adequate for our study, diagnostic or functional impairment studies are preferred. This is because they would provide more accurate and reliable information on the prevalence of mental health illnesses.

### Limitations

The main limitation of this study is the generalization of the result because it was an online survey rather than the gold standard structured interview. Moreover, the study may not have included parents and children who do not have internet access in some remote areas of Oman. Another limitation is that most of the surveys were filled by parents, and such a proxy approach may not accurately reflect the spectrum of depressive symptoms in children and adolescents.

### Conclusion

The prevalence of depression in children and adolescents may rise during pandemics if quarantine and social distancing measures are strictly applied.

The findings in our review have significant implications despite having limitations. Depressive symptoms in children and young adolescents can be prevented at earlier stages of pandemics. This can be achieved by identifying risk factors and formulating psychological interventions for vulnerable groups.
